# T-Cell-Driven Immunopathology and Fibrotic Remodeling in Hypertrophic Cardiomyopathy: A Translational Scoping Review

**DOI:** 10.3390/cells15010061

**Published:** 2025-12-29

**Authors:** Antonio da Silva Menezes Junior, Henrique Lima de Oliveira, Khissya Beatryz Alves de Lima, Silvia Marçal Botelho, Isabela Jubé Wastowski

**Affiliations:** 1 Faculty of Medicine, Federal University of Goiás, Goiânia 74605-050, Goiás, Brazil; henrique.lima2@discente.ufg.br (H.L.d.O.); khissya_beatryz@discente.ufg.br (K.B.A.d.L.); silviamarcal@ufg.br (S.M.B.); 2 School of Medical Sciences and Life, Pontifical Catholic University of Goiás, Goiânia 74605-050, Goiás, Brazil; 3 Immunology Department, State University of Goiás, Goiânia 74605-220, Goiás, Brazil; wastowski@gmail.com

**Keywords:** hypertrophic cardiomyopathy, immunogenetics, long noncoding RNA, RNA methylation, immune remodeling, precision medicine

## Abstract

**Highlights:**

**What are the main findings?**

- Hypertrophic cardiomyopathy (HCM) involves coordinated genetic, epigenetic, and immune remodeling, redefining it as an immunogenetic disorder rather than solely a sarcomeric disease.
- Key molecular drivers and immune cell shifts link RNA regulation, m6A methylation, and inflammatory pathways to myocardial fibrosis and diastolic dysfunction.

**What are the implications of the main finding?**

- Identified diagnostic gene panels and hub genes support transcriptome-based precision diagnostics for HCM.
- Immunometabolic drug targets and integration of biomarkers with imaging may guide personalized immunotherapy and risk stratification.

**Abstract:**

**Background**: Hypertrophic cardiomyopathy (HCM) is increasingly recognized as a disorder shaped not only by sarcomeric mutations but also by complex immunogenetic and metabolic interactions. Emerging transcriptomic and single-cell analyses implicate immune dysregulation, RNA methylation, and necroptosis as critical modulators of myocardial remodeling. **Objectives**: This scoping review synthesizes bioinformatic, transcriptomic, and experimental data to delineate the immunogenetic architecture of HCM and identify candidate molecular targets for immune–metabolic modulation. **Methods**: Following Joanna Briggs Institute and PRISMA-ScR guidelines, we systematically searched PubMed, Embase, Web of Science, and GEO through September 2025 for studies evaluating immune infiltration, RNA regulation, and necroptosis in human HCM. Data were narratively synthesized across histologic, clinical, and multi-omics domains. **Results**: Among 8191 screened records, 25 studies met the inclusion criteria. Key immune–epigenetic regulators included the lncRNA–mRNA pair MIR210HG–BPIFC, m6A readers IGFBP3 and YTHDC1, and necroptosis gene JAK2. The HCM myocardium exhibited the depletion of reparative M2 macrophages and Tregs; enrichment of cytotoxic CD8^+^ T cells; and activation of the TNFα–NFκB, IL-6–JAK–STAT3, and PI3K–Akt pathways. Machine learning biomarkers (RASD1, FCN3, and PIK3R1) exhibited diagnostic accuracy (AUC > 0.85). Drug target predictions identified ruxolitinib and celecoxib as potential immunometabolic modulators (agents predicted to modulate both immune and metabolic pathways based on gene expression signatures). **Conclusions**: These findings support a hypothesis that HCM may involve immunogenetic mechanisms, rather than being exclusively sarcomeric in nature, although this remains to be validated. The integration of molecular and imaging biomarkers may enable precision immunotherapy, redefining HCM from a structural cardiomyopathy to a biologically stratified condition.

## 1. Introduction

Hypertrophic cardiomyopathy (HCM) is the most common form of inherited cardiomyopathy. It remains one of the principal causes of heart failure, atrial fibrillation, and sudden cardiac death in young and apparently healthy individuals [1,2,3]. Although traditionally defined as a primary disease of the sarcomere driven predominantly by pathogenic variants in *MYBPC3* and *MYH7*, the clinical expression of HCM is remarkably heterogeneous, ranging from asymptomatic carriers to patients who present with progressive heart failure or malignant ventricular arrhythmias [4,5]. This heterogeneity cannot be explained solely by genetic factors. Indeed, even within families carrying the same variant, penetrance and phenotype vary considerably, suggesting the presence of additional molecular and environmental factors that influence disease trajectory [6,7].

Among these factors, immune activation and inflammation have recently been recognized as potential modifiers of HCM pathophysiology. It is well established that T cells play a key role in regulating myocardial remodeling in other forms of cardiovascular disease. Effector subsets, including cytotoxic CD8^+^ and pro-inflammatory CD4^+^ cells such as Th1 and Th17 cells, drive fibroblast proliferation and extracellular matrix expansion. In contrast, regulatory T cells (Tregs) counterbalance these processes by dampening inflammation and restraining maladaptive fibrosis [8,9,10]. The dynamic equilibrium between these opposing arms of the immune response appears to shape outcomes in ischemic and non-ischemic cardiomyopathies, suggesting that similar mechanisms may contribute to disease progression in HCM [11,12].

Evidence supporting this hypothesis is gradually increasing. Histological studies, although limited in scale, have consistently demonstrated lymphocytic infiltrates in the regions of myocardial disarray and fibrosis. Recent immunophenotyping studies have suggested that the balance between effector T cells and Tregs may be correlated with the extent of remodeling [13,14]. Clinical observations further support this concept: elevated circulating cytokine levels, including interleukin-6 and tumor necrosis factor-α, have been associated with diffuse myocardial fibrosis, as measured by cardiac magnetic resonance imaging, and with the occurrence of atrial and ventricular arrhythmias [15,16,17]. Moreover, transcriptomic and single-cell analyses have begun to delineate immune-fibrotic signaling networks, revealing the recurrent enrichment of pathways such as NF-κB, JAK/STAT, and interleukin-17 (IL-17) in myocardial samples from patients with HCM [18,19].

Most studies are small, use heterogeneous definitions of immune activation, and employ diverse endpoints, ranging from descriptive pathology to exploratory prognostic analyses. Consequently, it is difficult to ascertain whether T-cell activation in HCM is the primary driver of remodeling, a secondary bystander phenomenon, or a potential therapeutic target. Therefore, this scoping review aims to systematically map existing evidence linking immune and epigenetic mechanisms in HCM, identify consistent molecular mediators, and propose translational implications for precision therapy.

## 2. Methods

This scoping review was designed to systematically map current evidence describing immune, genetic, and epigenetic mechanisms in hypertrophic cardiomyopathy (HCM). This review followed the Joanna Briggs Institute (JBI) methodological framework for scoping reviews. It adhered to the Preferred Reporting Items for Systematic Reviews and Meta-Analyses extension for Scoping Reviews (PRISMA-ScR) guidelines, as shown in Supplementary Methods S1. A detailed protocol outlining the objectives, inclusion criteria, and analytic approach was prospectively registered on the Open Science Framework (OSF): doi.10.17605/OSF.IO/3NTUR.

### 2.1. Search Strategy

A comprehensive search strategy was developed in consultation with an experienced medical information specialist to capture the full breadth of the literature on immune and transcriptomic mechanisms in HCM. The following databases were searched from inception to 5 November 2025: PubMed/MEDLINE; Embase; Web of Science Core Collection; Cochrane Library; Gene Expression Omnibus (GEO) for transcriptomic datasets [20,21].

Search strings combined controlled vocabulary (MeSH and Emtree terms) with free-text keywords related to hypertrophic cardiomyopathy, T lymphocytes, macrophages, cytokines, fibrosis, epigenetics, lncRNA, m6A methylation, and immune infiltration. The complete search syntax for each database is provided in the Supplementary Materials to ensure reproducibility, as shown in Supplemental Methods S2.

### 2.2. Eligibility Criteria

Eligible studies met all the following criteria:

1. Population: Human participants diagnosed with HCM confirmed by imaging, histopathology, or genotyping.
2. Concept: Evaluation of immune activation, T-cell or macrophage subsets, cytokine expression, RNA modification, or necroptosis mechanisms.
3. Context: Investigations using histological, clinical, bioinformatic, transcriptomic, or single-cell approaches.
4. Publication characteristics: Peer-reviewed articles published in English.

We excluded the following: (i) animal or cell-only studies without human validation; (ii) case reports or series with fewer than five participants; (iii) conference abstracts, editorials, and gray literature; and (iv) studies without primary or integrative data on immune or molecular mechanisms.

### 2.3. Study Selection

All records were imported into EndNote X25 (Clarivate Analytics, 70 St Mary Axe, London, UK) for deduplication, then uploaded to Rayyan QCRI for blinded screening. Two independent reviewers screened titles and abstracts and evaluated the full texts of all potentially eligible studies. Discrepancies were resolved by discussion or consultation with a third senior reviewer.

### 2.4. Data Extraction

A standardized extraction template was piloted before implementation. The following data were systematically collected:

1. Bibliographic details (author, year, and country);
2. Study design and sample characteristics;
3. Assessment modality (immunohistochemistry, flow cytometry, RNA-seq, single-cell profiling, or bioinformatics);
4. Key immune or molecular findings (T-cell subsets, macrophage polarization, cytokine pathways, lncRNA–mRNA pairs, m6A readers, and necroptosis genes);
5. Clinical correlates (fibrosis, arrhythmia, or outcomes);
6. Main conclusions and limitations.

Data were independently extracted by two reviewers and cross-checked for accuracy.

### 2.5. Critical Appraisal of Evidence

Given the heterogeneity of study designs, a formal meta-analysis was not feasible. Instead, quality appraisal was conducted using validated instruments appropriate for each study type: AMSTAR 2 for systematic reviews; JBI critical appraisal tools for observational and biomarker studies; and evaluation of reproducibility, external validation, and biological plausibility for omics and single-cell datasets. Each study was categorized as having low, moderate, or high methodological strength, as shown in Supplemental Materials (Supplemental Tables S1A, S1B and S2).

### 2.6. Data Synthesis and Integration

The findings were synthesized narratively across four thematic domains:

1. Histologic and biopsy-based studies;
2. Clinical and biomarker cohorts;
3. Transcriptomic and bioinformatic analyses;
4. Translational and single-cell investigations.

To ensure analytical depth, recurring molecular pathways and immune signatures were tabulated and cross-validated across datasets. When available, GEO dataset identifiers (e.g., GSE36961 and GSE141910) were annotated to facilitate reproducibility. Molecular interactions and drug–target networks were further explored using *STRING*, *Enrichr*, and *DrugBank* databases to identify therapeutic candidates for immune–metabolic modulation.

### 2.7. Ethical Considerations

This study synthesized publicly available data and previously published reports; therefore, institutional ethics approval and informed consent were not required.

## 3. Results

**Figure 1:**
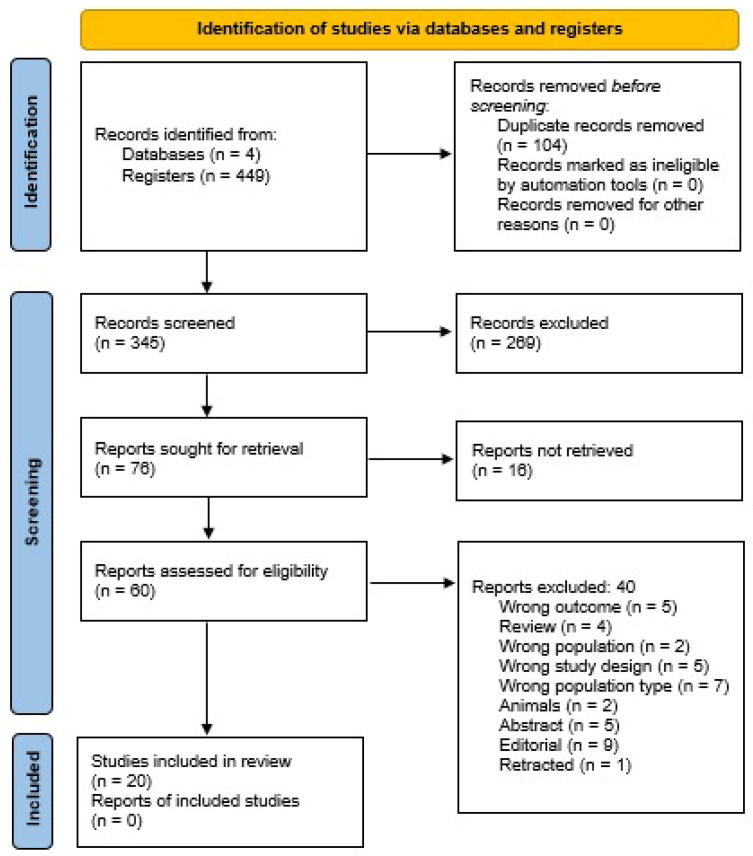
PRISMA Flowchart.

A total of 8191 prospective studies were identified in our scoping review, and 380 were included in the full-text review (Figure 1). A total of 25 studies were included in the analysis [22,23,24,25,26,27,28,29,30,31,32,33,34,35,36,37,38,39,40,41,42]. Following the analysis of the primary findings, we categorized the studies into several groups based on the research’s main features and results, as shown in Table 1.

### 3.1. Immune Dysregulation in Hypertrophic Cardiomyopathy

Recent studies using transcriptomic and bioinformatics analyses have identified a significant role for the immune system in the pathogenesis of HCM. The drug targets identified by network pharmacology lack sufficient dose–response data, on-target proof in HCM models, or safety assessments for the heart when used in combination with standard treatment, as shown in Supplementary Table S1A,B.

Specifically, changes in macrophage populations and T-cell subsets, along with the dysregulation of genes such as IGFBP3 and the lncRNA-mRNA pair MIR210HG-BPIFC, are central to these findings [22,23,24,25,26].

### 3.2. Immune–Epigenetic Crosstalk and Immune Cell Infiltration in Hypertrophic Cardiomyopathy

Recent transcriptomic and single-cell studies have uncovered a complex interplay between immune dysregulation and epigenetic regulation in hypertrophic cardiomyopathy (HCM). A central feature of the immune landscape in HCM is the marked imbalance of macrophage populations within the myocardium. Tissue analyses consistently show a significant reduction in LYVE1^+^CD163^+^ M2 macrophages, which are essential for maintaining tissue homeostasis and resolving inflammation, accompanied by an increase in pro-inflammatory M1 macrophages [25]. This macrophage polarization shift favors chronic inflammation and fibrotic remodeling, creating a microenvironment that promotes disease progression.

In parallel, T-cell dysregulation has emerged as another hallmark of the HCM immune profile. A notable reduction in naïve CD4^+^ T cells, together with an increase in cytotoxic CD8^+^ T cells, suggests an imbalance in adaptive immune activation [22]. Interestingly, the MIR210HG–BPIFC lncRNA–mRNA pair, which is downregulated in HCM, appears to modulate this process by influencing the infiltration and differentiation of these T-cell subsets [22]. This finding supports the notion that epigenetic control at the RNA level contributes to the remodeling of the myocardial immune microenvironment.

Comprehensive immune infiltration analyses further demonstrate a decreased representation of monocytes, dendritic cells, Th1 cells, and regulatory T cells (Tregs), indicating impaired adaptive immune surveillance in the hypertrophied myocardium [25]. Conversely, the increased infiltration of basophils and activated macrophages indicates sustained activation of the innate immune system, which perpetuates inflammatory signaling and contributes to interstitial fibrosis. Collectively, these cellular shifts depict an immune ecosystem skewed toward inflammation and tissue injury rather than repair.

**Figure 2:**
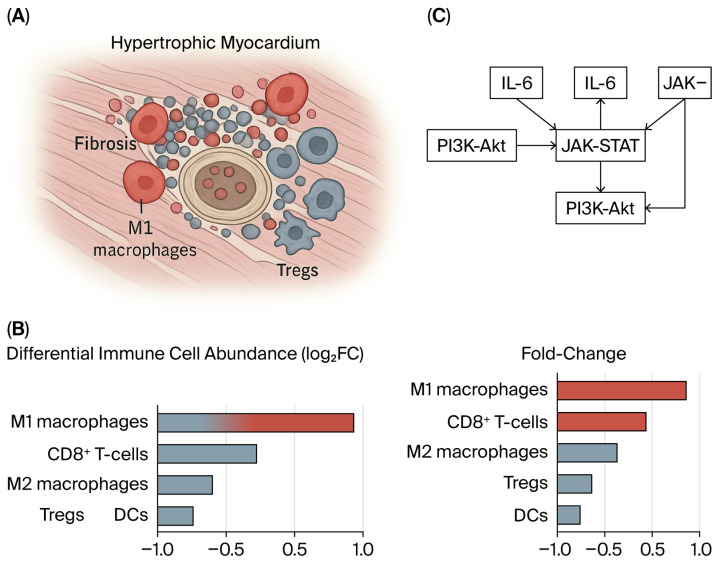
Immune cell remodeling in hypertrophic cardiomyopathy (HCM). (**A**) Schematic representation of hypertrophic myocardium showing immune infiltration and microstructural remodeling, characterized by increased M1 macrophages and CD8^+^ T-cells, reduced T regulatory cells (Tregs), and relative depletion of M2 macrophages and dendritic cells (DCs). Fibrotic deposition and myocyte disarray are illustrated as hallmarks of HCM pathology. (**B**) Differential abundance of immune cell populations (log_2_ fold-change), demonstrating up-regulation of pro-inflammatory M1 macrophages and CD8^+^ T-cells, and down-regulation of reparative M2 macrophages, Tregs, and DCs in HCM myocardium. (**C**) Key inflammatory signaling pathways implicated in immune-mediated myocardial remodeling, including IL-6–mediated activation of the JAK–STAT and PI3K–Akt pathways, contribute to fibrotic and hypertrophic responses. Abbreviations: DCs—Dendritic cells; HCM—Hypertrophic cardiomyopathy; IL-6—Interleukin-6; JAK—Janus kinase; PI3K—Phosphoinositide 3-kinase; STAT—Signal transducer and activator of transcription; Tregs—Regulatory T cells (CD4^+^CD25^+^FoxP3^+^).

**Figure 3:**
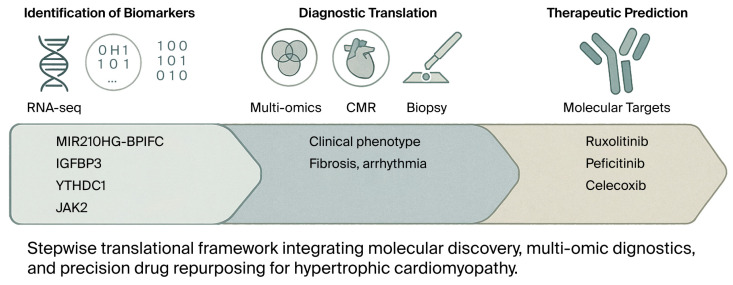
From mechanistic insight to precision immunotherapy in hypertrophic cardiomyopathy (HCM). This figure illustrates a stepwise translational framework connecting molecular discovery, diagnostic integration, and therapeutic prediction in HCM. Identification of biomarkers begins with RNA-seq and multi-omic analyses, highlighting key immunogenetic drivers, including *MIR210HG–BPIFC*, *IGFBP3*, *YTHDC1*, and *JAK2*. These candidate biomarkers undergo diagnostic translation using multi-omic profiling, cardiovascular magnetic resonance (CMR), and myocardial biopsy to link molecular signatures with clinical phenotypes, including fibrosis and arrhythmia susceptibility. Finally, therapeutic prediction leverages these mechanistic insights to identify potential immunomodulatory drug targets. It proposes repurposed agents—such as ruxolitinib, peficitinib, and celecoxib—to modulate the JAK–STAT and inflammatory pathways implicated in HCM remodeling. Overall, the figure summarizes an integrated precision-medicine pipeline for advancing targeted immunotherapy in hypertrophic cardiomyopathy. Abbreviations: CMR—Cardiovascular magnetic resonance; HCM—Hypertrophic cardiomyopathy; RNA-seq—RNA sequencing.

At the molecular level, epigenetic regulators are increasingly recognized as key orchestrators of this inflammatory milieu. The elevated expression of the m6A reader IGFBP3 has been linked to a highly enriched inflammatory state, marked by the increased infiltration of activated dendritic cells, macrophages, mast cells, and Tregs [23]. Pathway enrichment analyses revealed significant activation of TNFα signaling via NFκB and the IL6–JAK–STAT3 pathways in the high-IGFBP3 subgroup, underscoring the role of RNA methylation in amplifying cytokine-driven inflammatory remodeling [23] as shown in Figure 2 and Figure 3.

Balancing transcriptomic data identified a cluster of downregulated hub genes with critical roles in immune function, including *CD14*, *ITGB2*, *C1QB*, *CD163*, *HCLS1*, *ALOX5AP*, *PLEK*, *C1QC*, *FCER1G*, and *TYROBP*. These genes are involved in pathways governing inflammatory and innate immune responses and JAK–STAT signaling [24,25,26]. In addition, the necroptosis pathway, a regulated form of programmed cell death, is significantly enriched and activated in HCM myocardial tissue. Core necroptosis-related genes, such as CYBB, BCL2, and JAK2, show strong correlations with M2 macrophage infiltration, linking maladaptive cell death mechanisms to immune remodeling [24].

Together, these findings suggest that immune–epigenetic interactions form a critical axis in the pathophysiology of HCM. Epigenetic modifications, including m6A methylation and lncRNA-mediated regulation, appear to shape immune cell composition and function, thereby perpetuating chronic inflammation and fibrotic transformation.

Bioinformatic analyses corroborate these insights, consistently showing that HCM is characterized by upregulated innate immune activity—including the expansion of M0 macrophages (They refer to undifferentiated monocytes before polarization into M1 or M2 phenotype), monocytes, and natural killer (NK) cells—and the downregulation of adaptive immune regulatory components, such as M2 macrophages, Tregs, and plasma cells. This immunological imbalance favors a pro-inflammatory and pro-fibrotic myocardial environment that underpins disease progression and clinical heterogeneity, as summarized in Table 2.

### 3.3. Macrophage Heterogeneity and Remodeling in HCM

Macrophages play a central role in the immunopathology of hypertrophic cardiomyopathy (HCM), linking inflammation, fibrosis, and myocardial remodeling. Bioinformatic analyses of myocardial transcriptomes consistently reveal a profound imbalance between macrophage subtypes, characterized by a loss of reparative LYVE1^+^CD163^+^ M2 macrophages and an accumulation of pro-inflammatory M1 macrophages [25]. This polarization shift undermines tissue homeostasis and promotes fibrotic progression. Further, the absence of GATA3^+^ macrophages has been associated with improved myocardial function, suggesting that macrophage transcriptional phenotypes directly influence disease severity [28].

The differentiation of circulating monocytes into M1 and M2 macrophages appears to be dysregulated in HCM, shaping both inflammatory intensity and repair potential [27]. Interestingly, the results from different transcriptomic datasets are heterogeneous. While GSE32453 suggests increased macrophage, NK cell, and monocyte infiltration, GSE36961 and GSE141910 report the opposite pattern, with decreased macrophages and monocytes but increased CD8^+^ T cells, basophils, and fibroblasts [25]. These contrasting results likely reflect stage-dependent immune remodeling, in which immune overactivation and suppression may coexist across different disease contexts.

Histologically, CD68^+^ macrophages are enriched in fibrotic and necrotic regions, coordinating debris clearance and repair responses [27]. The upregulation of IGFBP3, an m6A RNA-binding protein, further correlates with the increased infiltration of macrophages, mast cells, and dendritic cells, linking metabolic and epigenetic signaling to immune activation [23]. Collectively, these findings support the view that HCM is not solely a structural or genetic disorder but also an immunologically dynamic disease, in which macrophage plasticity critically shapes fibrosis, ventricular compliance, and clinical outcomes.

Contrasting immune infiltration results—some reporting increased, others decreased macrophage/monocyte abundance—may reflect differences in sampling (e.g., myectomy vs. biopsy), disease stage, bioinformatic pipelines, or batch correction strategies. These variations underscore the need for standardized immune deconvolution methods and longitudinal sampling to define the trajectory of immune remodeling in HCM.

## 4. Discussion

Our scoping review, drawing on recent bioinformatic and experimental studies, identifies convergent genetic, epigenetic, and immunoregulatory signals that reshape current thinking on hypertrophic cardiomyopathy (HCM). Key candidates include the lncRNA–mRNA pair MIR210HG–BPIFC, the m6A readers IGFBP3 and YTHDC1, and the necroptosis-related kinase JAK2—each of which is emerging as a potential biomarker and therapeutic target. Moving forward, priorities include in vivo validation, mechanistic dissection, and clinical translation to improve diagnosis, risk stratification, and therapy [22,23,24,35,36,37]. While animal-only studies were excluded, findings from mouse models and iPSC-derived cardiomyocytes have informed our understanding of fibrosis, inflammation, and sarcomeric disarray. These models offer mechanistic insight into immune–fibrotic interactions and should be considered complementary to human data.

### 4.1. Genetic and Epigenetic Modulators of Immune Remodeling

Accumulating evidence indicates that non-coding RNA networks modulate myocardial inflammation and remodeling. The MIR210HG–BPIFC pair is downregulated in HCM and may shape immune cell infiltration—notably naïve CD4^+^ and CD8^+^ T cells—within the myocardium, implicating RNA-level control in disease progression [22]. At the epigenetic level, the m6A readers IGFBP3 and YTHDC1 are upregulated in HCM and, respectively, differentiate cases from controls with high accuracy, linking RNA methylation to immune activation and energy metabolism/mitophagy [23]. Together, these signals define a molecular axis linking transcriptional regulation with immune tone and metabolic stress [22,23,35,38,39,40,41,42].

### 4.2. Immunoregulation and Cell Infiltration

Across datasets, JAK–STAT appears among the most perturbed immune pathways—often enriched among downregulated genes—underscoring its role in maintaining immune balance in cardiac tissue [25,32,33]. Additional inflammation-linked pathways (e.g., MAPK, PI3K–Akt) accompany shifts in immune composition, highlighting complex immune dysregulation in HCM [25,28]. Functionally, IGFBP3-high tissues show activated dendritic cells, macrophages, mast cells, and Tregs, and the enrichment of TNFα–NFκB and IL6–JAK–STAT3 signaling—consistent with an inflamed myocardial microenvironment [23]. JAK2 also sits at the intersection of immune activation and necroptosis, correlating with macrophage phenotypes and linking cell death programs to fibrotic remodeling [24].

### 4.3. Biomarkers for Diagnosis and Risk Stratification

Multiple analytic strategies converge on promising diagnostic signatures. Machine learning analyses identify RASD1, CDC42EP4, MYH6, and FCN3 as strong classifiers of HCM vs. controls [29]. Independent analyses highlight a 10-gene hub panel—*CD14*, *ITGB2*, *C1QB*, *CD163*, *HCLS1*, *ALOX5AP*, *PLEK*, *C1QC*, *FCER1G*, and *TYROBP*—that is downregulated in myocardial tissue [26]. Gong et al. report eight additional candidates (*FOS*, *CD86*, *CD68*, *BDNF*, *PIK3R1*, *PLEK*, *RAC2*, and *CCL2*), each with an AUC > 0.8, reinforcing the diagnostic robustness of their approach [27]. Beyond expression panels, IGFBP3 and YTHDC1 (m6A readers) discriminate HCM and may bridge metabolic and immune dysregulation; IGFBP3 and JAK2 also associate with altered energy metabolism, strengthening their pathobiological relevance [23,28]. Clinically, these markers could improve differential diagnosis—particularly from hypertensive LVH—and refine risk stratification [23].

### 4.4. Integrated Model of Ischemia–Immune Interactions Driving Fibrosis in Hypertrophic Cardiomyopathy

Emerging data suggest that immune-mediated remodeling is not an isolated process; instead, it likely interacts with established ischemic mechanisms that have long been associated with hypertrophic cardiomyopathy. HCM is distinguished by microvascular dysfunction, which is caused by small-vessel thickening, a reduced capacity for vasodilation, and extravascular compression during diastole, the period when coronary perfusion should ideally occur. These pathological features result in recurrent supply–demand imbalances, focal ischemia, and myocyte necrosis, which subsequently elicit reparative fibrosis and contribute to the arrhythmogenic substrate. Furthermore, ischemic injury may also catalyze secondary immune activation, thereby facilitating macrophage recruitment, fibroblast proliferation, and cytokine-mediated extracellular matrix deposition.

Consequently, microvascular ischemia and immune remodeling should be considered synergistic processes, rather than mutually exclusive ones. Chronic ischemia initiates cellular damage and establishes a pro-inflammatory environment, thereby exacerbating the immunogenetic pathways discussed herein. This integrated perspective facilitates a more thorough comprehension of the multifaceted origins of fibrosis and electrical instability in HCM [43,44,45].

### 4.5. Therapeutic Targeting and Drug Repurposing

The same nodes that organize immune–metabolic crosstalk may be actionable. Bioinformatic predictions nominate ruxolitinib (JAK1/2) for JAK2 and celecoxib (COX-2) for IGFBP3 as candidates for targeted intervention; peficitinib (JAK1/2/3) has also been proposed to modulate JAK-dependent inflammation [28,30]. Pathway-level targets such as PIK3R1 (PI3K–Akt) suggest additional levers to limit hypertrophy and adverse remodeling [27]. These leads, while preliminary, support a precision immunometabolic therapeutic strategy anchored in patient-specific molecular profiles [22,23,24].

### 4.6. Targeted Candidates and Rationale (Concise)

JAK2 → ruxolitinib/peficitinib: These suppress cytokine-driven inflammation and have the potential to blunt JAK-STAT-linked remodeling [28,30]. IGFBP3 → celecoxib: These predict interactions in drug–target networks and may influence inflammation/fibrosis interfaces and metabolic signaling [23,28]. IGFBP3 and JAK2 co-expression: It is reproducibly higher across HCM datasets (e.g., GSE36961, GSE89714), strengthening its disease relevance [23,28]. These represent theoretical candidates for future exploration but lack direct testing in HCM models or clinical safety data in this context.

### 4.7. Imaging–Molecular Integration

CMR-LGE provides a robust, noninvasive assessment of fibrosis but may miss a subset of patients with histologically extensive fibrosis. EMB-derived collagen area fraction (CAF) and CD3^+^ T-cell counts independently predict adverse outcomes, and a combined high CAF/high CD3^+^ portends the worst prognosis [31]. Pairing CMR with genetic/epigenetic biomarkers (e.g., IGFBP3, YTHDC1, JAK2) could enhance phenotype discrimination and outcome prediction. At the same time, spatial transcriptomics suggests focal alterations in interferon signaling, mitochondrial metabolism, and ECM within disorganized regions that may be therapeutically exploitable [24,32,33].

### 4.8. Methodological Considerations When Aggregating Public Datasets

Leveraging multiple GEO and related datasets increases power, enables external validation, and improves model construction (e.g., integrating imaging features with clinical scores). Still, it introduces risks of dataset inconsistency, class imbalance, and overfitting. Findings must be confirmed in independent cohorts and, ultimately, via experimental validation in clinical samples and patient-derived cardiomyocytes [22,26,29,30,34].

Findings must be interpreted with caution, as several key analyses are derived from recurring GEO datasets (e.g., GSE36961, GSE141910). This may give the appearance of convergence, while in fact representing reanalyses of overlapping patient material. As such, it reduces the adequate sample size and limits the generalizability of findings across diverse cohorts.

### 4.9. Consolidated Outlook

Collectively, the evidence supports a genetic–epigenetic–immune axis in HCM: MIR210HG–BPIFC, IGFBP3, YTHDC1, and JAK2 link RNA regulation, immune activation, necroptosis, and metabolism, while hub and ML-derived genes (e.g., *CD14*, *ITGB2*, *C1QB*, *CD163*, *FOS*, *PIK3R1*, *RAC2*) expand the diagnostic toolkit [26,27,29]. The predicted agents (ruxolitinib, celecoxib, and peficitinib) exemplify a shift toward targeted immunometabolic therapy [28,30]. The next step is prospective, multimodal validation—melding omics, imaging, and pathology—to deliver molecularly guided diagnosis and treatment in HCM [22,23,24], as shown in Figure 3. The new evidence and clinical implications are presented in Supplemental Material Table S3.

### 4.10. Limitations

The current study has several limitations that should guide interpretation. This is a scoping synthesis that maps signals instead of counting them. Because there is no formal meta-analysis, we cannot estimate the effect size, quantify heterogeneity, or assess small-study and publication biases. Study identification was limited to peer-reviewed English-language publications, excluding non-English and gray sources that could skew biomarker results. There is substantial variety in the evidence base. The datasets include both bulk and single-cell platforms, including distinct preprocessing, normalization, and immune-deconvolution methods; cross-platform batch effects may be misinterpreted as biological concordance. GEO cohorts are often reanalyzed across different publications, which can create a false impression of reproducibility and effectively reduce the sample size. Tissue collection is variable (e.g., myectomy specimens, endomyocardial biopsy, and explant), and clinical confounders—such as genotype, disease stage, obstruction status, comorbidities, and concurrent cardiometabolic or disease-modifying therapies—are inconsistently recorded or managed. Most research is cross-sectional, which renders it difficult to draw causal conclusions. Different immune cell signals across datasets show how sensitive findings are to platform and cohort makeup. Translational assertions are still in their early stages. Pathway enrichment (e.g., IL-6/JAK-STAT, NF-κB, PI3K-Akt, and necroptosis) is mostly inferred in silico, supplemented with limited protein-level and phospho-signaling validation. Reported machine-learning biomarkers demonstrate promising discriminative performance, but without stringent external prospective validation, calibration, or decision-curve analysis. The drug targets identified by network pharmacology lack sufficient dose–response data, on-target proof in HCM models, or safety assessments for the heart when used with standard treatment. Lastly, molecular imaging integration is limited by the lack of studies combining patient-level omics with CMR (LGE/ECV), strain, or clinical outcomes. Moreover, there are too few pediatric cases, phenocopies, and individuals of diverse ancestries to make the results broadly generalizable. These limitations underscore the need for prospective, multicenter studies with standardized methodologies, high spatial resolution, protein-level validation, and early-phase, mechanism-informed trials before clinical implementation.

## 5. Conclusions

These findings support a hypothesis that HCM may involve immunogenetic mechanisms, rather than being exclusively sarcomeric in nature, although this remains to be validated. The convergence of evidence around MIR210HG–BPIFC, IGFBP3, YTHDC1, and JAK2 underscores an integrated axis linking RNA methylation, immune activation, necroptosis, and fibrosis. These molecules, together with downregulated or upregulated immune hub genes, form a promising foundation for biomarker-based diagnosis and targeted intervention.

Predicted pharmacologic modulators—such as ruxolitinib and celecoxib—illustrate the emerging feasibility of precision immunometabolic therapy in HCM. Future research should focus on validating these pathways in patient-derived cardiomyocytes, integrating imaging and molecular signatures, and testing targeted therapies in translational trials. Ultimately, this integrative framework bridges immunology, genetics, and therapeutics, offering a pathway toward individualized management and improved patient outcomes in hypertrophic cardiomyopathy. These predicted pharmacologic modulators, such as ruxolitinib and celecoxib, illustrate the theoretical potential of precision immunometabolic therapy but require rigorous preclinical evaluation.

## Figures and Tables

**Table 1:** Demographic characteristics of selected studies.

Author (Year)	Country/ Setting	Study Design	Population (*n*)	T-Cell/Marker Evaluated	Evaluation Method	Key Immunologic/ Molecular Findings	Cytokine/Pathway Associations	Fibrosis/Remodeling Evidence	Arrhythmia/ Clinical Outcomes	Level of Evidence/ Notes
Ellims (2012) [42]	Australia: tertiary CMR center	Observational comparative	51 HCM/25 controls	—(indirect immune readout)	CMR T1 mapping, LGE	↑ Diffuse interstitial fibrosis correlates with diastolic dysfunction and LA size	Indirect activation of IL-6/JAK-STAT & NF-κB via fibrosis	Fibrosis burden is tightly linked to LVH/ECV	No arrhythmic endpoints (risk inferred)	3—Imaging fibrosis; no immune phenotyping
Fang (2013) [38]	Australia; CMR + cell assays	Prospective observational	37 HCM/20 controls	Circulating fibrocyte precursors (PBMC → fibrocyte)	Post-contrast T1; fibrocyte culture/flow	Lower T1 (↑ fibrosis) associates with ↑ fibrocyte differentiation; CXCL12 ↑	CXCL12/SDF-1 chemokine axis; leukocyte trafficking	Fibrocyte activity inversely correlates with T1 (ECM expansion)	Diastolic dysfunction association; no rhythm outcomes	3—Small; immune readout peripheral
Tanaka 2014 [40]	Japan/Germany; academic labs	Translational model	iPSC-CM (3 HCM/3 ctrl); Mybpc3 KI mice	—(ET-1 trigger; myocyte disarray)	iPSC-CM stimulation; motion vector; mouse validation	ET-1 induces hypertrophy/disarray; ETA blockade protective	ET-1/ETA axis; downstream MAPK	Reproduces structural disarray phenotype	Arrhythmogenic substrate inferred by contractile variability	3—In vitro/model; no patient outcomes
Helms 2016 [37]	USA; myectomy tissue	Translational tissue case–control	25 genotype+/10 genotype−/8 ctrl	CaMKII/PLN signaling (Ca^2+^ handling)	Protein assays; Ca^2+^ uptake	CaMKII activation in sarcomere-mutant HCM; SERCA2 ↓	CaMKII–PLN (pT17); no calcineurin/NFAT	Links Ca^2+^ mishandling to hypertrophy/ECM	Mechanistic link to arrhythmia substrate; no clinical events	2–3—Strong tissue mechanistics
Kalyva 2016 [39]	Greece; tertiary center	Prospective observational	40 HCM/23 controls	EPCs (CD45−/CD34+/VEGFR2+, CD133+)	Flow cytometry	↑ EPCs; correlate with E/e′ (diastolic dysfunction)	VEGF/angiogenesis; endothelial injury	Microvascular dysfunction ↔ fibrosis (indirect)	Not arrhythmia-focused	3—Immune-vascular axis; modest n
Shintani 2022 [31]	Japan, university hospital	Clinical–histological	34 HCM biopsies	TGF-β1, CTGF, COL1A1, α-SMA (with macrophages)	IHC; RT-PCR	Co-localization of TGF-β1/CTGF with fibrotic areas & macrophage infiltrates	TGF-β/SMAD profibrotic signaling	Fibrosis proportional to TGF-β1 expression	Fibrosis severity associated with ventricular arrhythmia burden	3—Single center; no causality
Wu 2022 [26]	China/Saudi; bioinfo	Cross-sectional bioinformatics	GEO GSE36961 (106 HCM/39 ctrl)	Immune hub genes (CD14, ITGB2, CD163, TYROBP…)	DEG; PPI; ceRNA network	Immune-activation signature implicating leukocyte pathways	Complement/coagulation; NF-κB	ECM organization genes up	No outcomes (diagnostic modeling)	4—In silico; no wet-lab
Li 2022 [23]	China; multi-omics	Bioinformatic + in vitro	GSE36961/130036; H9c2 cells	IGFBP3 (m6A-related), YTHDC1	RF/LASSO/WGCNA; qPCR	IGFBP3 ↑ (immune-ECM); YTHDC1 → mitophagy (PINK1-PRKN)	TNFα/NF-κB; IL-6–JAK–STAT3	IGFBP3 overexpression → ↑ COL1A2/COL3A1/MMP9	No clinical outcomes	3–4—Cell validation; no tissue outcomes
Laird 2023 [32]	USA; myectomy tissue	Spatial transcriptomics	HCM *n* = 4/donor *n* = 2	Region-specific immune/ECM programs	GeoMx panel; snRNA-seq	Severe disarray regions: ↑ mitochondrial/ECM; ↓ interferon signaling	PDGF, fibronectin, CD99, and APP networks	↑ Fibroblast/vascular composition in disarray	Not powered for outcomes	3—High-res, small n
You & Dong 2023 [29]	China; ML-bioinfo	Diagnostic bioinformatics	GSE36961; GSE141910	RASD1, CDC42EP4, MYH6, FCN3	LASSO; SVM-RFE; PPI	Four-gene diagnostic panel; AUC > 0.9	Inflammatory response; complement; VEGFA–VEGFR2; apoptosis	ECM regulatory links	No outcomes	4—Secondary datasets; ML focus
Gong 2024 [27]	China; multi-dataset	Bioinformatic + qPCR	GSE32453 & GSE36961	Immune infiltration (macrophages, NK, CD4 memory)	CIBERSORT; PPI; qPCR	Hub genes FOS, CD86, CD68, BDNF linked to immune-fibrotic signature	MAPK; PI3K-Akt; NF-κB	Immune infiltration tracks ECM genes	Arrhythmic substrate inferred	4—Limited validation
Zhang 2021 [25]	China	RNA-seq re-analysis + in vitro	GSE180313; GSE130036; AC16 cells	lncRNA MIR210HG–BPIFC; CD8^+^/naïve CD4^+^ signatures	WGCNA; ceRNA; qPCR	MIR210HG–BPIFC down → ↑ CD8^+^, ↓ naïve CD4^+^	IL-6, TNF-α cytokine–lncRNA interplay	Immune-ECM linkage (inferred)	—	3–4—Mechanism inferred; small validation
Marketou 2015 [36]	Greece; biomarker study	Prospective cohort	54 HCM/40 controls	IL-6, TNF-α, IL-17A; endothelial function	ELISA; echo	↑ IL-6/TNF-α associated with LVH & diastolic dysfunction	Systemic inflammation; endothelial-immune crosstalk	Cytokines associate with microvascular impairment/fibrosis	Trend to higher arrhythmic risk with cytokine load	3—Systemic markers; no long FU
Hou 2024 [24]	China; discovery/validation	Bioinformatic immune-necroptosis	GSE130036; GSE141910	Necroptosis-related genes (CYBB, BCL2, JAK2)	NRDEG; PPI/CytoHubba; CIBERSORT	Necroptosis signature activated in HCM	HIF-1; NOD-like receptor; inflammatory signaling	Links to ECM/inflammation	Diagnostic ROC only	4—In silico; needs tissue validation
Zhang 2024 [22]	China; RNA Seq	Bioinformatics (RNA-Seq) and in vitro validation	HCM Patients: 13 (GSE180313) + 28 (GSE130036) Healthy Controls: 7 (GSE180313) + 9 (GSE130036)	Naive CD4^+^ T cells and CD8^+^ T cells	RNA-Seq, qRT-PCR, CIBERSORTx	Immune cell infiltration analysis	CD8^+^ T cells inreased and naive CD4^+^ T cells decreased resting Mast cells decreased	Fibrosis grade mirrors proteomic signature	LV mass & diastolic indices correlate	3—Tissue–plasma translational link
Zhuo 2025 [30]	China	Integrative bioinfo + wet-lab	GSE36961; GSE141910; NRCM	JAK2, EDNRA, KCNA5, DNAJC15, CA3, PRKCD, KLF2	LASSO/SVM-RFE; qRT-PCR/IF	Oxidative-stress gene panel → apoptosis & immune shift	ROS; apoptosis; cytokine signaling	OS-DEGs correlate with ECM remodeling	In vitro confirmation; no outcomes	3–4—Robust pipeline; no patient outcomes
Cai 2025 [28]	China; systems bioinfo	WGCNA + ML with validation	GSE36961; GSE89714	IGFBP3, JAK2 (energy-immune cross-talk)	WGCNA; LASSO/SVM-RFE; ssGSEA	Energy-metabolism signature with immune skew	IL-6–JAK–STAT; insulin resistance	Network ties to ECM/metabolic remodeling	Drug predictions: ruxolitinib, celecoxib	4—Hypothesis-generating
Cao 2025 [35]	China/USA; pooled RNA-seq	Meta-analysis of RNA-seq	9 datasets (109 HCM/210 ctrl)	ST2/IL1RL1; immune deconvolution	DESeq2; CIBERSORTx; PPI	ST2 markedly down; broad inflammatory dysregulation	Correlations: IL-6, CD163; ↓ Tregs, ↑ neutrophils	ST2 network tied to profibrotic responses	No outcomes	3–4—Consistent cross-dataset signal
Wang 2025 [33]	China; national consortium	Integrative multi-omics + qPCR	GSE36961; GSE141910; qRT-PCR (16/16)	**Tregs**: FOXP3, IL2RA, CTLA4	WGCNA; RF/LASSO; CIBERSORTx; docking	Treg-associated biomarkers are reduced in HCM	IL-2/STAT5; NF-κB; TGF-β	Treg ↓ links to ECM genes (COL1A1, FN1, MMP2)	Indirect arrhythmic susceptibility	3–4—Translational signal; no longitudinal data
He 2025 [34]	China; Single-center study.	Bidirectional Mendelian Randomization (MR)	108 patients with HCM	Eff-memory CD4^+^/CD8^+^, Tregs (protective) vs. CD8dim, CCR2^+^ DCs (risk)	IVW; MR-Egger; sensitivity	31 immune cell types causally linked to HCM	IL-2/Treg axis; immune activation	Inferred immune-driven remodeling	No functional outcomes	1–2—Causal inference; needs tissue proof
Ali 2025 [41]	USA; multi-species	Translational single-nuclei	Human *n* = 5; feline *n* = 7; mouse *n* = 4	Immune/non-CM clusters; fibroblast crosstalk	snRNA-seq; GO enrichment	Conserved hypertrophy/energy programs; immune heterogeneity	Calcium/sarcomeric; oxidative metabolism	ECM/fibrosis pathways enriched in non-CM	Not assessed	2—High-resolution; cross-species

Abbreviations: AF, atrial fibrillation; CMR, cardiac magnetic resonance; CTGF, connective tissue growth factor; DC, dendritic cell; DEG, differentially expressed genes; ECM, extracellular matrix; ECV, extracellular volume; EPC, endothelial progenitor cell; ETA, endothelin A receptor; FU, follow-up; GSEA, gene set enrichment analysis; GWAS, genome-wide association study; HCM, hypertrophic cardiomyopathy; Imunometabolic therapy (It opposes substances that, according to gene expression signatures, are anticipated to alter metabolic and immune pathways); IHC, immunohistochemistry; iPSC-CM, induced pluripotent stem cell–derived cardiomyocytes; JAHA, Journal of the American Heart Association; JAK/STAT, Janus kinase/signal transducer and activator of transcription; KI, knock-in; LGE, late gadolinium enhancement; L-R, ligand–receptor; LVH, left ventricular hypertrophy; ML, machine learning; MR, Mendelian randomization; NF-κB, nuclear factor kappa B; NRCM, neonatal rat cardiomyocyte; PBMC, peripheral blood mononuclear cells; PPI, protein–protein interaction; qPCR/qRT-PCR, (quantitative) real-time polymerase chain reaction; RF, random forest; scRNA-seq/snRNA-seq, single-cell/single-nucleus RNA sequencing; SDF-1, stromal cell–derived factor 1 (CXCL12); ssGSEA, single-sample gene set enrichment analysis; ST2/IL1RL1, suppressor of tumorigenicity 2; TGF-β, transforming growth factor-beta; TIL, tumor-infiltrating lymphocyte; Treg, regulatory T cell; WGCNA, weighted gene co-expression network analysis. ↑: increase; ↓: decrease; →: leads to; ↔: related to.

**Table 2:** Integrated overview of immune infiltration, molecular pathways, and translational biomarkers in HCM.

Category	Key Molecules/ Genes	Associated Immune Cells/Pathways	Main Findings	Biological/ Clinical Implications	Dataset (GEO/GSE)	Supporting Studies
1. m6A RNA Methylation Regulators	IGFBP3, YTHDC1	Increase in activated dendritic cells, macrophages, mast cells, Tregs; *TNFα–NFκB*, *IL6–JAK–STAT3* signaling	IGFBP3 upregulation correlates with inflammatory and angiogenic gene signatures; YTHDC1 is linked to mitophagy suppression and metabolic dysregulation	Epigenetic m6A modifications amplify immune and fibrotic remodeling; potential m6A-targeted therapies.	GSE36961/GSE89714	Li 2022 [23]; Cai 2025 [28]
2. lncRNA–mRNA Regulatory Pair	MIR210HG–BPIFC	Decrease in naïve CD4^+^ T cells; Increase in CD8^+^ T cells	Downregulation disrupts T-cell balance and promotes cytotoxic infiltration in HCM myocardium	lncRNA network dysregulation modulates immune microenvironment; potential diagnostic target	GSE141910/GSE32453	Zhang 2024 [22]
3. Necroptosis-Related Genes	CYBB, BCL2, JAK2	Correlated with M2 macrophage infiltration; *TNF*, *IL-17*, *JAK–STAT* signaling	Necroptosis pathways are enriched in HCM; an imbalance between cell death and repair promotes fibrosis.	Targeting the necroptosis–macrophage axis may reduce inflammatory remodeling.	GSE36961	Hou 2024 [24]
4. Downregulated Hub Genes	CD14, ITGB2, C1QB, CD163, HCLS1, ALOX5AP, PLEK, C1QC, FCER1G, TYROBP	Innate immune and complement activation pathways	Reduced expression indicates impaired immune surveillance and macrophage polarization.	Early diagnostic markers and targets for anti-inflammatory therapies	GSE141910/GSE36961	Wu 2022 [26]; Zhang 2021 [25]
5. Immune Infiltration Patterns	—	Increase in M0 macrophages, monocytes, NK cells; Decrease in M2 macrophages, Tregs, Th1 cells	CIBERSORT and ssGSEA analyses demonstrate an immune imbalance toward pro-inflammatory phenotypes	Chronic innate activation drives fibrosis and arrhythmogenic remodeling	GSE32453/GSE36961/GSE141910	Zhang 2021 [25]; Gong 2024 [27]
6. Machine-Learning-Derived Diagnostic Genes	RASD1, CDC42EP4, MYH6, FCN3	Immune-modulatory and structural signaling networks	High AUC (>0.85) for differentiating HCM from controls in ML models	Promising biomarkers for computational diagnostic screening	GSE36961/GSE141910	You & Dong 2023 [29]
7. Therapeutic Targets (Predicted)	IGFBP3, JAK2, YTHDC1, CYBB, BCL2	*JAK–STAT* and *COX-2* signaling	*Ruxolitinib*, *peficitinib* (JAK inhibitors), and *celecoxib* (COX-2 modulator) are predicted to target core pathways.	Drug repurposing offers precision immunometabolic therapy options	GSE36961/GSE89714	Cai 2025 [28]; Zhuo 2025 [30]
8. Imaging–Molecular Correlation	CD3^+^ T cells, CAF (collagen area fraction)	Fibrosis and inflammation quantified by EMB vs. CMR	High CAF + CD3^+^ T-cell infiltration predicts poor outcomes even without LGE on CMR	Combining molecular and imaging markers enhances risk stratification and diagnostic accuracy	—	Shintani 2022 [31]; Laird 2023 [32]

## Data Availability

No new data were created or analyzed in this study. Data sharing does not apply to this study.

## Supplementary Materials

The following supporting information can be downloaded at: https://www.mdpi.com/article/10.3390/cells15010061/s1, Methods S1: PRISMA; Methods S2: Search Strategy; Table S1A: Characteristics of included studies evaluating T-cell immunity in hypertrophic cardiomyopathy (HCM); Table S1B: Characteristics of included studies evaluating T-cell immunity in hypertrophic cardiomyopathy (HCM); Table S2: Study Appraisal Summary (JBI Framework); Table S3: Translational Perspective.

## Abbreviations

AF	Atrial fibrillation
ALOX5AP	Arachidonate 5-lipoxygenase-activating protein
AUC	Area under the curve
BCL2	B-cell lymphoma 2
BDNF	Brain-derived neurotrophic factor
BPIFC	Bactericidal/permeability-increasing fold-containing family C protein
CAF	Collagen area fraction
CD	Cluster of differentiation
CD14	Cluster of differentiation 14
CD68	Cluster of differentiation 68
CD86	Cluster of differentiation 86
CDC42EP4	CDC42 effector protein 4
C1QB	Complement component 1, q subcomponent, B chain
C1QC	Complement component 1, q subcomponent, C chain
CMR	Cardiac magnetic resonance
CYBB	Cytochrome b-245, beta chain
DC	Dendritic cell
ECM	Extracellular matrix
FCER1G	Fc fragment of IgE receptor Ig
FCN3	Ficolin-3
FOS	Fos proto-oncogene, AP-1 transcription factor subunit
GEO	Gene Expression Omnibus
HCM	Hypertrophic cardiomyopathy
HOCM	Hypertrophic obstructive cardiomyopathy
HCLS1	Hematopoietic cell-specific Lyn substrate 1
IGFBP3	Insulin-like growth factor-binding protein 3
IL	Interleukin
IL-6	Interleukin-6
IL-17	Interleukin-17
INF-γ	Interferon-gamma
ITGB2	Integrin subunit beta 2
JAK	Janus kinase
JAK-STAT	Janus kinase/signal transducer and activator of transcription
LGE	Late gadolinium enhancement
lncRNA	Long noncoding RNA
LV	Left ventricle
LVEF	Left ventricular ejection fraction
LYVE1	Lymphatic vessel endothelial hyaluronan receptor 1
M0/M1/M2	Macrophage phenotypes (naïve/pro-inflammatory/reparative)
MAPK	Mitogen-activated protein kinase
m6A	N6-methyladenosine
ML	Machine learning
mRNA	Messenger RNA
MSC	Mesenchymal stem cell
MYBPC3	Myosin-binding protein C, cardiac type
MYH6	Myosin heavy chain 6
MYH7	Myosin heavy chain 7
NF-κB	Nuclear factor kappa B
NK	Natural killer cell
PI3K–Akt	Phosphoinositide 3-kinase–protein kinase B
PIK3R1	Phosphoinositide-3-kinase regulatory subunit 1
PLEK	Pleckstrin
PRISMA-ScR	Preferred Reporting Items for Systematic Reviews and Meta-Analyses extension for Scoping Reviews
RASD1	Ras-related dexamethasone-induced 1
RNA-seq	RNA sequencing
scRNA-seq	Single-cell RNA sequencing
STAT3	Signal transducer and activator of transcription 3
ST2	Suppression of tumorigenicity 2 (IL-33 receptor)
TGF-β	Transforming growth factor beta
Th	T helper
TNF-α	Tumor necrosis factor alpha
Treg	Regulatory T cell
TYROBP	TYRO protein tyrosine kinase-binding protein
WGCNA	Weighted gene co-expression network analysis
YTHDC1	YTH domain-containing protein 1
