# Supplementary material for: T-Cell-Driven Immunopathology and Fibrotic Remodeling in Hypertrophic Cardiomyopathy: A Translational Scoping Review
Source: Cells. 2025 Dec 29;15(1):61. doi: 10.3390/cells15010061 (PMC12785778; doi:10.3390/cells15010061)
Supplement: Supplementary file 1 [file cells-15-00061-s001.zip › cells-4021854-supplementary.pdf]

Supplementary Material

T-cell–Driven Immunopathology and Fibrotic Remodeling in Hypertrophic Cardiomyopathy: A Translational Scoping Review

Antonio da Silva Menezes Junior [1,2,*], Henrique Lima de Oliveira [1], Khissya Beatryz Alves de Lima [1], Silvia Marçal Botelho [1,2] and Isabela Jubé Wastowski [3]

1 Faculty of Medicine, Federal University of Goiás, Goiânia 74605-050, Goiás, Brazil

2 School of Medical Sciences and Life, Pontifical Catholic University of Goiás, Goiânia 74605-050, Goiás, Brazil

3 Immunology Department, State University of Goiás, Goiânia 74605-220, Goiás, Brazil

* Correspondence: a.menezes.junior@uol.com.br; Tel.: +55-629-8271-1177

Summary

Supplemental Methods 1. PRISMA Checklist: 3

Supplemental Methods 2. Search Strategy 5

Supplemental Table S1A. Characteristics of included studies evaluating T-cell immunity in hypertrophic cardiomyopathy (HCM) 6

Supplemental Table S1B. Characteristics of included studies evaluating T-cell immunity in hypertrophic cardiomyopathy (HCM) 8

Supplemental Table S2. Study Appraisal Summary (JBI Framework) 11

Supplemental Table S3. Translational Perspective: 14

Supplemental Methods 1. PRISMA Checklist:

SECTION	ITEM	PRISMA-ScR CHECKLIST ITEM	REPORTED ON PAGE #
**TITLE**			
Title	1	Identify the report as a scoping review.	1
**ABSTRACT**			
Structured summary	2	Provide a structured summary that includes (as applicable): background, objectives, eligibility criteria, sources of evidence, charting methods, results, and conclusions that relate to the review questions and objectives.	1-2
**INTRODUCTION**			
Rationale	3	Describe the rationale for the review in the context of what is already known. Explain why the review questions/objectives lend themselves to a scoping review approach.	2
Objectives	4	Provide an explicit statement of the questions and objectives being addressed with reference to their key elements (e.g., population or participants, concepts, and context) or other relevant key elements used to conceptualize the review questions and/or objectives.	2
**METHODS**			
Protocol and registration	5	Indicate whether a review protocol exists; state if and where it can be accessed (e.g., a Web address); and if available, provide registration information, including the registration number.	3 https://osf.io/ts8n4
Eligibility criteria	6	Specify characteristics of the sources of evidence used as eligibility criteria (e.g., years considered, language, and publication status), and provide a rationale.	3-4
Information sources*	7	Describe all information sources in the search (e.g., databases with dates of coverage and contact with authors to identify additional sources), as well as the date the most recent search was executed.	3-4
Search	8	Present the full electronic search strategy for at least 1 database, including any limits used, such that it could be repeated.	3
Selection of sources of evidence†	9	State the process for selecting sources of evidence (i.e., screening and eligibility) included in the scoping review.	3-5
Data charting process‡	10	Describe the methods of charting data from the included sources of evidence (e.g., calibrated forms or forms that have been tested by the team before their use, and whether data charting was done independently or in duplicate) and any processes for obtaining and confirming data from investigators.	4
Data items	11	List and define all variables for which data were sought and any assumptions and simplifications made.	4
Critical appraisal of individual sources of evidence§	12	If done, provide a rationale for conducting a critical appraisal of included sources of evidence; describe the methods used and how this information was used in any data synthesis (if appropriate).	4
Synthesis of results	13	Describe the methods of handling and summarizing the data that were charted.	4
**RESULTS**			

SECTION	ITEM	PRISMA-ScR CHECKLIST ITEM	REPORTED ON PAGE #
Selection of sources of evidence	14	Give numbers of sources of evidence screened, assessed for eligibility, and included in the review, with reasons for exclusions at each stage, ideally using a flow diagram.	4
Characteristics of sources of evidence	15	For each source of evidence, present characteristics for which data were charted and provide the citations.	4 and table 1
Critical appraisal within sources of evidence	16	If done, present data on critical appraisal of included sources of evidence (see item 12).	4-13
Results of individual sources of evidence	17	For each included source of evidence, present the relevant data that were charted that relate to the review questions and objectives.	4-13
Synthesis of results	18	Summarize and/or present the charting results as they relate to the review questions and objectives.	13-15
**DISCUSSION**			
Summary of evidence	19	Summarize the main results (including an overview of concepts, themes, and types of evidence available), link to the review questions and objectives, and consider the relevance to key groups.	13
Limitations	20	Discuss the limitations of the scoping review process.	13-14
Conclusions	21	Provide a general interpretation of the results with respect to the review questions and objectives, as well as potential implications and/or next steps.	14-15
**FUNDING**			
Funding	22	Describe sources of funding for the included sources of evidence, as well as sources of funding for the scoping review. Describe the role of the funders of the scoping review.	NA

Supplemental Methods 2. Search Strategy

Database	Search Strategy
**PubMed (MEDLINE)**	("Hypertrophic Cardiomyopathy"[Mesh] OR "hypertrophic cardiomyopathy"[tiab] OR hypertrophic cardiomyopath*[tiab] OR HCM[tiab] OR "hypertrophic obstructive cardiomyopathy"[tiab] OR HOCM[tiab]) AND ("T-Lymphocytes"[Mesh] OR T-lymphocyte*[tiab] OR "T cell*"[tiab] OR T-cell*[tiab] OR "regulatory T cell*"[tiab] OR Treg*[tiab] OR CD4[tiab] OR CD8[tiab] OR Th1[tiab] OR Th17[tiab] OR Tfh[tiab]) AND (fibrosis[tiab] OR remodeling[tiab] OR inflammation[tiab] OR immune[tiab] OR cytokine*[tiab] OR diagnos*[tiab] OR sensitivity[tiab] OR specificity[tiab] OR AUC[tiab] OR ROC[tiab] OR prognos*[tiab] OR predict*[tiab] OR hazard*[tiab] OR outcome*[tiab] OR risk[tiab] OR survival[tiab] OR arrhythmia*[tiab] OR "atrial fibrillation"[tiab] OR AF[tiab] OR "sudden cardiac death"[tiab] OR SCD[tiab] OR ICD[tiab]) NOT (animals[mh] NOT humans[mh]) = **41**
**Embase (Elsevier/Ovid)**	('hypertrophic cardiomyopathy'/exp OR hypertrophic cardiomyopath*:ti,ab,kw OR hocm:ti,ab,kw OR hcm:ti,ab,kw) AND ('t lymphocyte'/exp OR 't cell'/exp OR 'regulatory t lymphocyte'/exp OR 't cell*':ti,ab,kw OR t-lymphocyte*:ti,ab,kw OR treg*:ti,ab,kw OR cd4*:ti,ab,kw OR cd8*:ti,ab,kw OR th1:ti,ab,kw OR th17:ti,ab,kw OR tfh:ti,ab,kw) AND (fibrosis:ti,ab,kw OR remodeling:ti,ab,kw OR inflammation:ti,ab,kw OR immune*:ti,ab,kw OR cytokine*:ti,ab,kw OR diagnos*:ti,ab,kw OR sensitivity:ti,ab,kw OR specificity:ti,ab,kw OR auc:ti,ab,kw OR roc:ti,ab,kw OR prognos*:ti,ab,kw OR predict*:ti,ab,kw OR hazard*:ti,ab,kw OR risk:ti,ab,kw OR survival:ti,ab,kw OR arrhythmia*:ti,ab,kw OR 'atrial fibrillation':ti,ab,kw OR af:ti,ab,kw OR 'sudden cardiac death':ti,ab,kw OR scd:ti,ab,kw OR icd:ti,ab,kw) = **197**
**Cochrane Library (CENTRAL / Reviews)**	([mh "Hypertrophic Cardiomyopathy"] OR hypertrophic cardiomyopathy:ti,ab,kw OR hypertrophic cardiomyopath*:ti,ab,kw OR HCM:ti,ab,kw OR HOCM:ti,ab,kw) AND ([mh "T-Lymphocytes"] OR T cell*:ti,ab,kw OR T lymphocyte*:ti,ab,kw OR Treg*:ti,ab,kw OR CD4:ti,ab,kw OR CD8:ti,ab,kw OR Th1:ti,ab,kw OR Th17:ti,ab,kw OR Tfh:ti,ab,kw) AND (fibrosis:ti,ab,kw OR remodeling:ti,ab,kw OR inflammation:ti,ab,kw OR immune:ti,ab,kw OR cytokine*:ti,ab,kw OR diagnos*:ti,ab,kw OR prognos*:ti,ab,kw OR predict*:ti,ab,kw OR outcome*:ti,ab,kw OR risk:ti,ab,kw OR survival:ti,ab,kw OR arrhythmia*:ti,ab,kw OR "atrial fibrillation":ti,ab,kw OR AF:ti,ab,kw OR "sudden cardiac death":ti,ab,kw OR SCD:ti,ab,kw OR ICD:ti,ab,kw) : **5**
**Scopus**	TITLE-ABS-KEY(("hypertrophic cardiomyopathy" OR hypertrophic cardiomyopath* OR HCM OR "hypertrophic obstructive cardiomyopathy" OR HOCM) AND ("T cell*" OR "T lymphocyte*" OR T-lymphocyte* OR Treg* OR "regulatory T cell*" OR CD4 OR CD8 OR Th1 OR Th17 OR Tfh) AND (fibrosis OR remodeling OR inflammation OR immune OR immunology OR cytokine* OR diagnos* OR sensitivity OR specificity OR "area under the curve" OR ROC OR AUC OR prognos* OR predict* OR outcome* OR risk OR survival OR arrhythmia* OR "atrial fibrillation" OR AF OR "sudden cardiac death" OR SCD OR ICD)) : **206**

Supplemental Table S1A. Characteristics of included studies evaluating T-cell immunity in hypertrophic cardiomyopathy (HCM)

Author [Ref.]	Year	Study Type / Modality	Datasets / Cohorts	Key Biomarkers / Pathways
**Helms AS et al. [37]**	2016	Human myocardium; transcriptomics; calcium signaling	Institutional cohort	Ca^{2+} signaling perturbations
**Marketou ME et al. [36]**	2015	Flow cytometry; peripheral blood	Single-center HCM vs controls	$CD45^{-}$; $CD34^{-}$; $CD90^{+}$ mesenchymal stem cells (MSCs)
**Laird J et al. [32]**	2023	Spatial transcriptomics; human myocardium	Human HCM surgical samples	Interferon signaling; extracellular matrix (ECM); mitochondrial metabolism
**Li Y et al. [23]**	2022	Bulk RNA-seq; bioinformatics; validation	GEO (e.g., GSE130036)	*IGFBP3*; *YTHDC1*; m6A modification; mitophagy
**Hou J et al. [24]**	2024	Bulk RNA-seq; bioinformatics	GEO (e.g., GSE141910)	*CYBB*; *BCL2*; *JAK2*; necroptosis
**Gong J et. al. [27]**	2024	Bulk RNA-seq; bioinformatics; machine learning (AUC)	GSE33453; GSE36961	*FOS*; *CDK6*; *RAC2*; *IL2*; PI3K–Akt; MAPK signaling
**Zhuo et al. [30]**	2025	Integrative bioinformatics with in vitro validation	Discovery: GSE36961; Validation: GSE141910; neonatal rat cardiomyocytes	*JAK2*; *EDNRA*; *KCNA5*; *DNAJC15*; *CA3*; *PRKCD*; *KLF2*; JAK–STAT; MAPK; TGF-β; NF-κB; apoptosis; mitochondrial and immune signaling
**Wu S et al. [26]**	2022	WGCNA; hub-gene network	GEO (GSE130036)	*CD14*; *ITGB2*; *C1QB*; *CD163*; *HCLS1*; *ALOX5AP*; *RAC2*; *CCL2*; MAPK; PI3K–Akt
**You H et al. [29]**	2023	Integrated machine-learning diagnostic modeling	GEO (training and validation cohorts)	*RASD1*; *CDC42EP4*; *MYH6*; *FCN3*

**Tanaka T et al. [40]**	2014	Cardiac magnetic resonance; LGE; prognosis	Single-center cohort	Late gadolinium enhancement (LGE) extent
**Fang L et al. [38]**	2013	CMR T1 mapping; circulating fibrocytes	HCM vs controls	Fibrocytes; native T1 mapping
**Shintani Y et al. [31]**	2022	Endomyocardial biopsy; histopathology	Clinical cohort	Cancer-associated fibroblasts (CAFs); $CD3^{+}$ T cells
**Cao X et al. [35]**	2025	Meta-analysis; transcriptomics; immune deconvolution	Integrated cohorts	ST2 (*IL1RL1*); inflammatory remodeling
**Cai S et. al. [28]**	2025	Bioinformatics; metabolic analysis	GSE36961; GSE89741	*IGFBP3*; *JAK2*; immune–metabolic pathways
**Wang J et al. [33]**	2025	Multi-omics analysis	Public datasets with validation	Immune–metabolic cross-talk
**Ali AS et al. [41]**	2025	Single-cell RNA-seq; cross-species analysis	Human and experimental models	Conserved immune–fibrotic programs; variable gene expression
**Ellims AH et al. [42]**	2012	Prospective CMR cohort	HCM patients and healthy controls	Native T1; ECV; LGE; LV mass index; strain; extracellular matrix expansion
**Kalyva A et al. [39]**	2016	Peripheral blood gene expression	Single-center HCM vs controls	CD45; CD34; CD90; mesenchymal stem cells
**He X et al. [34]**	2025	Two-sample bidirectional Mendelian randomization	GWAS of 731 immune traits + HCM GWAS	Protective: Tregs; HLA-DR^{+} cells; monocytes. Risk: $CD4^{+}CD8$^dim T cells; $CD62L^{-}$ dendritic cells. Pathways: antigen presentation; T-cell regulation
**Zhang Y et al. [22]**	2024	Integrated transcriptomic analysis	GSE180313; GSE130036; ISO-induced AC16 cardiomyocyte model	MIR210HG; BPIFC; miR-145; miR-216b; miR-24; miR-34c; ceRNA networks; T-cell infiltration; JAK2–STAT3; RhoA; TGF-β/Smad

Abbreviations: AAV: adeno-associated virus; AC16: human ventricular cardiomyocyte cell line; AUC: area under the curve; CAF: cancer-associated fibroblast; Ca^{2+}: calcium ion; CCL2: C-C motif chemokine ligand 2; CD: cluster of differentiation; ceRNA: competing endogenous RNA; CMR: cardiac magnetic resonance; ECM: extracellular matrix; FCN3: ficolin-3; GEO: Gene Expression Omnibus; GWAS: genome-wide association study; HCM: hypertrophic cardiomyopathy; HCLS1: hematopoietic cell-specific Lyn substrate 1; HLA-DR: human leukocyte antigen-DR isotype; IGFBP3: insulin-like growth factor–binding protein 3; IL-2: interleukin-2; ISO: isoproterenol; ITGB2: integrin beta-2; JAK2: Janus kinase 2; LGE: late gadolinium enhancement; lncRNA: long non-coding RNA; MAPK: mitogen-activated protein kinase; m6A: N6-methyladenosine; miRNA: microRNA; ML: machine learning; mRNA: messenger RNA; MSC: mesenchymal stem cell; MYBPC3: myosin-binding protein C; NK: natural killer cell; PI3K: phosphoinositide 3-kinase; RASD1: Ras dexamethasone-induced 1; RNA-seq: RNA sequencing; RhoA: Ras homolog family member A; scRNA-seq: single-cell RNA sequencing; STAT3: signal transducer and activator of transcription 3; ST2 (IL-33R): interleukin-33 receptor; TGF-β: transforming growth factor beta; Treg: regulatory T cell; WGCNA: weighted gene co-expression network analysis; YTHDC1: YTH domain-containing protein 1; MR: Mendelian randomization; CD4+CD8dim: double-positive T-cell subset with dim CD8 expression; CD62L: L-selectin; MIR210HG: miR-210 host gene lncRNA; BPIFC: BPI fold-containing family C protein; miR-145: microRNA-145; miR-216b: microRNA-216b; miR-24: microRNA-24; miR-34c: microRNA-34c.

Supplemental Table S1B. Characteristics of included studies evaluating T-cell immunity in hypertrophic cardiomyopathy (HCM)

**Author [Ref.]**	**Year**	**Key Findings**	**Translational Implications**
Helms AS et al. [37]	2016	HCM exhibits genotype-dependent and genotype-independent Ca^{2+} dysregulation	Supports Ca^{2+}-targeted modulation irrespective of sarcomeric genotype
Kalyva A et al. [39]	2016	Altered cytokine and apoptotic gene expression in HCM	Peripheral immune signatures may assist non-invasive immune monitoring
Marketou ME et al. [36]	2015	Elevated circulating MSC-like cells correlate with LV mass index	Potential blood-based biomarker of myocardial remodeling
Laird J et al. [32]	2023	Myocardial disarray regions show distinct interferon, ECM, and metabolic signatures.	Spatially targeted anti-inflammatory and anti-fibrotic therapies
Li Y et al. [23]	2022	*IGFBP3* and *YTHDC1* upregulation linked to inflammation and mitophagy	Diagnostic potential; m6A regulatory axis as a therapeutic avenue
Hou J et al. [24]	2024	Necroptosis pathways enriched with associated immune imbalance	Necroptosis represents a candidate therapeutic target
Gong J et al. [27]	2024	Multi-gene immune and stress-response signature with diagnostic relevance	Supports machine–learning–based immune diagnostic stratification

Zhuo et al. [30]	2025	Identified 33 oxidative-stress-related DEGs; seven key markers (*JAK2*, *EDNRA*, *KCNA5*, *DNAJC15*, *CA3*, *PRKCD*, *KLF2*) showed strong diagnostic performance and were linked to apoptosis, immune dysregulation, and hypertrophic signaling; validated in independent datasets and HCM-like cardiomyocytes	Markers may support early diagnosis, risk stratification, and therapeutic targeting of oxidative stress, apoptosis, and immune remodeling in HCM.
Wu S et al. [26]	2022	Downregulated immune hub genes identified in HCM myocardium	Candidate diagnostic markers targeting immune modulation
You H et al. [29]	2023	High diagnostic performance of a four-gene panel	Supports precision diagnostic approaches
Tanaka T et al. [40]	2014	Late gadolinium enhancement (LGE) predicts adverse clinical outcomes	Improved risk stratification using cardiac magnetic resonance
Fang L et al. [38]	2013	Circulating fibrocytes correlate with myocardial fibrosis indices	Blood-based monitoring of fibrotic burden
Shintani Y et al. [31]	2022	Cancer-associated fibroblasts and $CD3^+$ T-cell counts independently predict outcomes.	Biopsy-based risk stratification and immune profiling
Cao X et al. [35]	2025	Loss of ST2 (*IL1RL1*) signaling is associated with inflammatory remodeling	ST2/IL-33 axis as a potential therapeutic target
Cai S et al. [28]	2025	Energy-metabolic genes link immune dysregulation to myocardial remodeling	Drug repurposing candidates (e.g., ruxolitinib, celecoxib)
Wang J et al. [33]	2025	Convergent immune and metabolic pathways identified in HCM	Candidate targets for precision therapies
Ali AS et al. [41]	2025	Conserved biological pathways despite expression divergence across species	Guides model selection for preclinical and translational studies
Ellims AH et al. [42]	2012	Diffuse myocardial fibrosis, as assessed by native T1 mapping and ECV, correlates with diastolic dysfunction, LV hypertrophy, reduced strain, and global cardiac impairment.	T1 mapping and ECV as sensitive non-invasive biomarkers for fibrosis, risk stratification, and therapeutic monitoring

He X et al. [34]	2025	Thirty-one immune cell types are causally linked to HCM (19 protective, 12 risk); HCM also alters 18 immune subsets.	Immune profiles as biomarkers and targets for immunomodulatory therapy (e.g., Treg enhancement, suppression of pro-inflammatory cells)
Zhang Y et al. [22]	2024	MIR210HG–BPIFC axis consistently downregulated; MIR210HG knockdown reduces *BPIFC*, increases inflammatory RNAs, and induces cardiomyocyte hypertrophy	MIR210HG–BPIFC as a novel biomarker and therapeutic target via lncRNA/miRNA modulation

Supplemental Table S2. Study Appraisal Summary (JBI Framework)

Study (Author, Year)	**Type of Study**	**Appraisal Tool**	**Evidence Level**	**Justification**
Helms AS, 2016 [37]	Human myocardium; transcriptomics; calcium signaling	JBI Checklist for Analytical Cross-Sectional Studies	Level 4	Bioinformatic or transcriptomic analyses are observational analytical designs.
Kalyva A, 2016 [39]	Peripheral blood gene expression	JBI Checklist for Analytical Studies	Level 4	Analytical design provides moderate evidence for exploratory research.
Marketou ME, 2015 [36]	Flow cytometry; peripheral blood	JBI Checklist for Analytical Studies	Level 4	Analytical design provides moderate evidence for exploratory research.
Laird J, 2023 [32]	Spatial transcriptomics; human myocardium	JBI Checklist for Analytical Cross-Sectional Studies	Level 4	Bioinformatic or transcriptomic analyses are observational analytical designs.
Li Y, 2022 [23]	Bulk RNA-seq bioinformatics; validation	JBI Checklist for Analytical Cross-Sectional Studies	Level 4	Bioinformatic or transcriptomic analyses are observational analytical designs.
Hou J, 2024 [24]	Bulk RNA-seq bioinformatics	JBI Checklist for Analytical Cross-Sectional Studies	Level 4	Bioinformatic or transcriptomic analyses are observational analytical designs.

Gong J, 2024 [27]	Bulk RNA-seq bioinformatics; ML AUC	JBI Checklist for Analytical Cross-Sectional Studies	Level 4	Bioinformatic or transcriptomic analyses are observational analytical designs.
Zhuo, 2025 [30]	Analytical observational study	JBI Checklist for Analytical Cross-Sectional Studies	Level 4	justified because it relies on secondary transcriptomic datasets without prospective clinical sampling.
HE, 2025 [34]	Bioinformatic / Mendelian Randomization	JBI Checklist for Analytical Cross-Sectional Studies	Level 4	Study uses GWAS-based two-sample bidirectional Mendelian Randomization; observational, secondary data analysis.
Wu S, 2022 [26]	WGCNA; hub gene network	JBI Checklist for Analytical Studies	Level 4	Analytical design provides moderate evidence for exploratory research.
You H, 2023 [29]	Integrated ML diagnostic modeling	JBI Checklist for Analytical Studies	Level 4	Analytical design provides moderate evidence for exploratory research.
Tanaka T, 2014 [40]	CMR LGE; prognosis	JBI Checklist for Analytical Studies	Level 4	Analytical design provides moderate evidence for exploratory research.
Fang L, 2013 [38]	CMR T1; circulating fibrocytes	JBI Checklist for Analytical Studies	Level 4	Analytical design provides moderate evidence for exploratory research.

Shintani Y, 2022 [31]	Endomyocardial biopsy; pathology	JBI Checklist for Analytical Studies	Level 4	Analytical design provides moderate evidence for exploratory research.
Zhang,2021 [22]	Bioinformatic + Experimental Validation	JBI Checklist for Analytical Cross-Sectional Studies	Level 3	Integrates transcriptomic datasets (RNA-seq), WGCNA, immune infiltration analysis, and in vitro validation in AC16 cardiomyocytes.
Cao X, 2025 [35]	Meta-analysis; transcriptomics; immune deconvolution	JBI Checklist for Analytical Cross-Sectional Studies	Level 4	Bioinformatic or transcriptomic analyses are observational analytical designs.
Cai S, 2025 [28]	Bioinformatics; metabolism	JBI Checklist for Analytical Cross-Sectional Studies	Level 4	Bioinformatic or transcriptomic analyses are observational analytical designs.
Wang J, 2025 [43]	Multi-omics	JBI Checklist for Analytical Studies	Level 4	Analytical design provides moderate evidence for exploratory research.
Ali AS, 2025 [41]	scRNA-seq; cross-species	JBI Checklist for Analytical Cross-Sectional Studies	Level 4	Bioinformatic or transcriptomic analyses are observational analytical designs.
Ellims,2012 [42]	Prospective imaging cohort study	JBI Checklist for Analytical Studies	Level 3	Observational prospective cohort using imaging biomarkers to correlate diffuse fibrosis (T1/ECV) with functional cardiac parameters; no randomization or intervention; high-quality diagnostic/imaging evidence..

Supplemental Table S3. Translational Perspective:

What Is New	Translational Relevance
• T-cell–mediated inflammation and regulatory imbalance actively remodel the hypertrophic myocardium.• Effector ($CD8^+$, Th17) activation and Treg depletion form a self-perpetuating immune-fibrotic loop.• This mechanism links sarcomeric stress, cytokine signaling (IL-6, IL-17A, TNF-α, TGF-β1), and extracellular-matrix expansion.	• Circulating immune biomarkers (IL-6, IL-17A, galectin-3, IGFBP3) and cardiac MRI indices (LGE, ECV) jointly reflect immune-fibrotic remodeling.• A low-cost, integrative immune–imaging profile enables early recognition of "immune-active" HCM.• This framework supports biomarker-driven diagnosis and monitoring across genotype-positive and genotype-negative patients.
Clinical Implications	Future Directions
• Combining immune and imaging metrics refines arrhythmic-risk prediction and patient selection for device or drug therapy.• Phenotyping by immune activity allows precision follow-up and trial enrollment based on biology rather than morphology.	• Therapeutic strategies aiming to restore Treg homeostasis or inhibit IL-6/JAK-STAT3 signaling may reverse fibrosis. Immune-guided precision phenotyping has the potential to transform HCM management from structural description to biologic modulation.
